# “He Will Not Leave Us Alone and I Need the Courts to Help”: Defendants’ Use of Nonphysical Violence in Domestic Violence Protective Order Cases

**DOI:** 10.1177/10778012221101921

**Published:** 2022-08-22

**Authors:** Erika M. Redding, Kathryn E. Moracco, Clare L. Barrington, Allyson M. Corbo

**Affiliations:** 1 Department of Health Behavior, Gillings School of Global Public Health, The University of North Carolina at Chapel Hill, Chapel Hill, NC, USA; 2 UNC Injury Prevention Research Center, The University of North Carolina at Chapel Hill, Chapel Hill, NC, USA; 3 6856 RTI International, Center for Communication Science, Research Triangle Park, NC, USA

**Keywords:** domestic violence, mental health and violence, legal intervention, battered women

## Abstract

District court judges who make final determinations in domestic violence protective order (DVPO) cases in North Carolina indicate often using heuristics, such as the presence of visible injury, to guide their assessment of violence severity. This approach is concerning as it minimizes nonphysical intimate partner violence. We conducted a thematic analysis of DVPO plaintiff complaints to identify the types of nonphysical vioence described and its effects on plaintiff health outcomes. Most case files included descriptions of nonphysical violence and plaintiffs described fear as a significant mental health outcome. Findings highlight the potentially deleterious impact of nonphysical violence on the well-being of DVPO plaintiffs.

## Introduction

Intimate partner violence (IPV) is categorized into four main types, including physical violence, sexual violence, stalking, and psychological aggression, and can be perpetrated by a current or former intimate partner (Centers for Disease Control and Prevention, n.d.). IPV is a persistent and widespread social harm; nearly 3,000 people in the United States are killed by current or former intimate partners every year and 36% of women and 11% of men report having been raped, physically assaulted, or stalked by an intimate partner in their lifetime (Smith et al., 2018). Experiencing IPV is linked to severe negative mental and physical health outcomes such as depression, substance use, chronic disease, and fatal and nonfatal injury (Coker et al., 2002; Smith et al., 2018). IPV survivors often require services from healthcare facilities as well as state-level and local domestic violence (DV) programs (Macy et al., 2009). Research also indicates that legal action, such as DV protective orders (DVPOs), is an efficacious method for survivors to seek protection after experiencing violence (Holt et al., 2002; Kothari et al., 2012; Logan & Walker, 2010; Logan et al., 2012). DVPOs are court-mandated civil orders prohibiting alleged assailants (defendants) from contacting victims (plaintiffs) for a specified period of time, usually 12 months (Dejong & Burgess-Proctor, 2006). IPV survivors who are granted a DVPO generally have less contact with their abusers, fewer police incidents, lower risk of IPV-related injuries, and fewer emergency department visits (Benitez et al., 2010; Carlson et al., 1999; Holt et al., 2003; Kothari et al., 2012).

**Figure 1.**
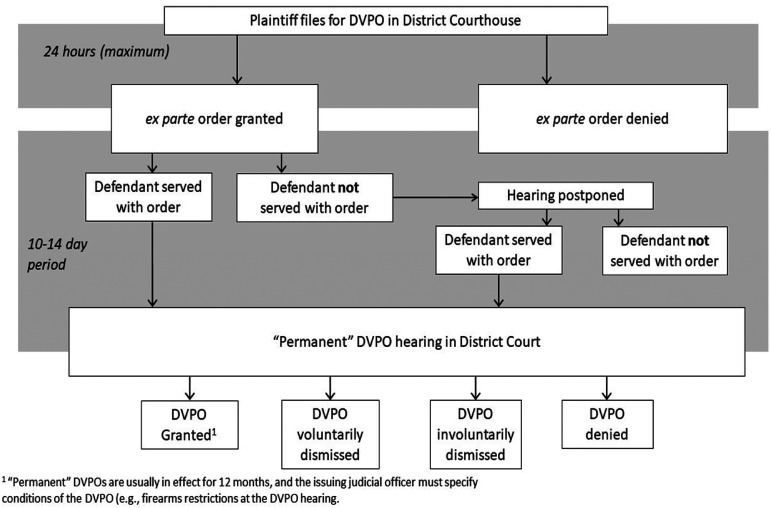
Domestic violence protective order (DVPO) process in North Carolina.

At the time that data for this study were collected, in North Carolina (NC) DVPOs could be granted for those who experienced violence within the following types of relationships: current or former spouses, persons of the opposite sex who live/have lived together, persons who are related as parents/guardians and children, persons who have a child in common, persons who are current or former household members, or persons of the opposite sex who are in a dating relationship/previously dated; however, the statute has since been amended to include same-sex partners (North Carolina General Assembly, 2005). When filing for a DVPO, plaintiffs must complete an initial complaint form that requests demographic and personal identification information about both the plaintiff and the defendant. This form also provides space for the plaintiff to write a short narrative detailing their experiences of violence (North Carolina General Assembly, 2005). This study will focus on data collected from these initial DVPO complaints, the entire DVPO process in NC is further depicted in Figure 1.

DVPO hearings are often placed on a crowded court docket where judges have limited time to review a plaintiff's initial complaints and hear the case (Agnew-Brune et al., 2017). As a result of these time constraints, judges may rely on a myriad of quick decision-making strategies. One such strategy is using physical injury such as bruising, cuts, scarring, etc., whether indicated in the DVPO complaint form or plaintiff testimony, as a gauge that violence has reached a perceived “threshold” of severity and imminent danger that warrants a DVPO (Agnew-Brune et al., 2017). For judges, evidence of physical injury can serve as a heuristic, or mental shortcut, allowing them to make what they perceive to be accurate decisions in a short amount of time (Agnew-Brune et al., 2017).

While physical injury is an important component of IPV, the NC statute also includes forms of nonphysical violence in its definition of IPV (North Carolina General Assembly, 2005). Nonphysical violence as defined by the NC statute includes, acts of stalking and harassment, and/or placing the plaintiff, their family, or a member of their household, “in fear of imminent serious bodily injury” that rises “to such a level as to inflict substantial emotional destress” (North Carolina General Assembly, 2005). Judges’ use of physical injury as an indication of IPV severity, therefore, poses substantial concerns as it diminishes the importance of nonphysical forms of IPV. Beyond the use of heuristics, previous studies also indicate that there are other ways that judges may systematically ignore the important role of nonphysical IPV in DVPO cases. In a 2019 study, researchers determined that some judges consider various types of nonphysical violence to be “frivolous” and attributed to circumstances that do not warrant a DVPO (Kafka et al., 2019). This lack of consideration could lead judges to overlook circumstances that are violent and linked to negative physical and mental health outcomes for survivors, including future abuse (Logan et al., 2006; O’Leary, 1999; Queen et al., 2009).

This study is a part of a larger project entitled *Courts Applying Solutions to End Intimate Partner Violence* (CASE IPV). CASE IPV seeks to describe how DVPO hearings are implemented across NC and to identify real-world practices that have the potential to improve DVPO processes and outcomes. The present study focuses on a qualitative analysis of initial DVPO complaint narratives from plaintiffs in three urban NC counties. These narratives are considered by judges when making decisions for ex parte orders (temporary protection until the DVPO hearing) and during the final DVPO hearing during which permanent DVPO determinations are made. We chose to focus on IPV victimization as described by urban women, as previous literature indicates that there may be distinct differences in experiences of IPV between urban and rural women (Edwards, 2015; Peek-Asa et al., 2011). The purpose of this study was to identify how nonphysical violence, as described by DVPO plaintiffs in NC, was used by defendants against their intimate partners. The research questions guiding this study were: (1) what types of nonphysical violence are described by plaintiffs in DVPO complaint narratives? and (2) how do plaintiffs describe the effects of nonphysical violence?

## Methods

### Data Collection

We analyzed 89 plaintiff narratives contained within the initial complaint of DVPO case files in NC. In NC, DVPO case file information is publicly available. Case files included in this study were from a representative sample of 406 DVPO case files collected as part of the parent study (CASE IPV), which was determined to be exempt from further review by the University of North Carolina at Chapel Hill institutional review board. The case files consist of paperwork for both granted and nongranted DVPOs that were filed between June 2016 and September 2017 and all narratives contain plaintiffs’ descriptions of the incident that initiated their pursuit of a DVPO. Our primary data source provides first-hand accounts regarding the alleged violence experienced by plaintiffs, and we were interested in considering the potential public health implications of these violent encounters. As previously mentioned, due to our interest in urban women and the distinct nature of their experiences with IPV, we included data from three NC counties that are home to some of the largest cities in the state (“U.S. Census Bureau QuickFacts: North Carolina,” n.d.). Additionally, these three counties have consistently high DVPO filing volumes as compared to others in the state (North Carolina Administrative Office of the Court, n.d.). As per the CASE IPV inclusion criteria, all narratives were written by plaintiffs who identified as female and were over the age of 18. In total 27, 18, and 44, narratives came from these three counties, for a total of 89 cases.

### Data Analysis

The first author (E.R.) and fourth author (A.C.) prepared the 89 plaintiff narratives for analysis, which included reading each narrative in its entirety and transcribing the content verbatim into a shared Microsoft Excel (Version 1910) document. Information regarding the hearing location (i.e., county and city), date of hearing, plaintiff, and defendant demographic information as described in the DVPO paperwork, DVPO hearing outcome, and the involvement of weapons in the case was also included in the Excel file. Data from the Excel file was then uploaded to ATLAS.ti (Version 8) for further analysis.

The data were analyzed using a thematic analytical approach, which Braun and Clarke (2006) describe as a process in which researchers systematically code for frequently emerging themes within a dataset to derive meaning and inform assertions (Braun & Clarke, 2006). Our epistemological position for this study was rooted in realism, in that we sought to authentically report and discuss the lived experiences of IPV survivors in their own words (Braun & Clarke, 2006). While our study aim was to highlight the experiences of survivors of IPV and to understand how they describe instances of nonphysical violence, each aspect of our analysis process was based on decisions made by the research team, and meaning was drawn from our own understanding and interpretations (Braun & Clarke, 2006). As scholars specializing in the field of IPV and violence prevention, our interpretations of these narratives are inherently informed by an understanding of the patriarchal societal structures that facilitate violence against women as well as in-depth knowledge regarding inequities imbedded within judicial systems.

Data analysis was conducted in iterative, overlapping phases, beginning with the first author reading through plaintiff narratives and taking detailed notes regarding salient themes, and identifying commonalities and differences. After this familiarization phase, the first author developed memos in which she interrogated her own epistemological stance and practiced reflexivity by identifying initial impressions and reactions to the data (Saldana, 2015). This process was useful in informing how she would begin to draw meaning from the narratives later in the analysis process (Saldana, 2015). Next, the first author developed a preliminary codebook that was shared with the second (K.M.), third (C.B.), and fourth authors, and the research team collaboratively edited and refined the codebook. This preliminary codebook contained topical codes generated from a priori knowledge regarding judges’ decision-making processes and accepted definitions of nonphysical violence as described by the Centers for Disease Control and Prevention and the NC statute, as well as interpretative codes generated from insights garnered through the initial reading of the DVPO narratives. Examples of topical codes included: “severity of physical violence,” “non-physical violence,” “plaintiff expression of fear,” “other parties involved in violence,” “timeline of violence,” “plaintiff reason for seeking order,” and “DVPO outcome.” Examples of interpretative codes included: “resilience,” “coercive control,” and “social support.”

Coded datum varied from short lines of text to entire narratives depending on the code applied. During the initial coding process, the first author paid particular attention to ensuring the inclusion of adequate text within each coded segment to contextualize the excerpt while simultaneously avoiding “word overload” or the inclusion of text that did not advance analysis (Miles & Huberman, 1984; Sandelowski, 1995). While the contextualization of each coded segment is important for analytical interpretations, “word overload” produces results that are “analytically and contextually empty” (Sandelowski, 1995). Beyond the application of codes to the narrative text, the first author conducted extensive memoing related to the themes and patterns within the data (Saldana, 2015).

Upon completion of this initial coding process, the first author conducted another round of coding and memoing to reduce data into categories. During this phase, the first author was particularly interested in synthesizing coded data into distinct categories. Next, the first author assessed the commonality of themes across code groups to assess potential relationships (Saldana, 2015). Finally, the first author conducted the last round of memoing during which time she began to organize and synthesize themes regarding nonphysical violence into distinct types as well as to theorize potential implications of violence for the well-being of plaintiffs. In addition to qualitative data analysis, our team conducted a descriptive analysis of plaintiff and defendant racial demographic data at the county level; while data regarding plaintiff racial identity and ethnic identity was not included in all case files, we were able to obtain plaintiff demographic information for 79 (88.8%) cases. We did not include gender-based demographic information in our analysis as only cases filed by adult women were included in this study. Additionally, other demographic indicators such as education, income, etc., are not consistently available within case file data. Beyond demographic information, we analyzed data regarding case disposition (i.e., whether a case was granted or denied) and the indicated involvement of weapons in case files. To obtain descriptive indicators of demographic and case outcome information, we stratified our data by county (*N* = 3) and calculated the percentage of cases that met the inclusion criteria for each category out of the total number of cases within the county.

## Results

In terms of racial diversity, across case files, over one-third of plaintiffs identified as being non-Hispanic White, and slightly over 40% of plaintiffs indicated that they identified as non-Hispanic Black, with a small proportion identifying as “other race” such as Asian or Pacific Islander. In terms of ethnicity, almost 10% of plaintiffs identified as Hispanic. Similar to plaintiff racial demographics, about one-third of plaintiffs identified as White and 40% identified as Black. Eight percent identified as “other race” and about 5% identified as Hispanic. Additionally, most cases (83%) included in our sample were granted and about one-fifth (20%) involved weapons. More detailed descriptive results, including county-level stratification of findings, can be found in Table 1.

**Table 1.** Domestic Violence Protection Order (DVPO) Case Information by County.

DVPO case information	County #1 *n* (%)	County #2 *n* (%)	County #3 *n* (%)	Sample total *N* (%)
*Plaintiff's race/ethnicity*				
Non-Hispanic White	6 (22.2)	7 (38.9)	20 (45.4)	33 (37.1)
Non-Hispanic Black	10 (37.0)	9 (50)	18 (40.9)	37 (41.6)
Hispanic	6 (22.2)	0	0	6 (6.7)
Other	2 (7.4)	0	1 (2.3)	3 (3.4)
Missing	3 (11.1)	2 (11.1)	5 (11.4)	10 (11.2)
*Defendant's race/ethnicity*				
Non-Hispanic White	10 (37.0)	8 (44.4)	16 (36.4)	34 (38.2)
Non-Hispanic Black	11 (40.7)	8 (44.4)	21 (47.7)	40 (45)
Hispanic	5 (18.5)	0	0	5 (5.6)
Other	1 (3.7)	1 (5.6)	6 (13.6)	8 (9)
Missing	0	1 (5.6)	1 (2.3)	2 (2.2)
*Case disposition*				
Granted	24 (88.9)	14 (77.8)	36 (81.8)	74 (83)
Denied	3 (11.1)	4 (22.2)	8 (18.2)	15 (17)
*Weapons involved*				
Yes	4 (14.8)	5 (27.8)	9 (20.5)	18 (20.2)
No	23 (85.2)	13 (72.2)	35 (79.5)	71 (79.8)

References to nonphysical violence were prevalent throughout the narratives; in fact, mentions of nonphysical violence occurred more frequently than mentions of physical violence. Within the 89 narratives, 65 (73.0%) contained descriptions of nonphysical violence compared to 56 (62.9%) that contained themes of physical violence; 35 (39.3%) narratives included descriptions of both physical and nonphysical violence; these trends underscore frequency with which nonphysical violence is often described by plaintiffs seeking a DVPO. In the sections that follow, we describe types of nonphysical violence as described by plaintiffs including harassment and stalking; degradation; and threats. Additionally, we consider the effects of nonphysical violence as described by plaintiffs. Demonstrative quotes included throughout this section are direct transcriptions of plaintiff narratives in the exact language in which they were written. Although case file information, including plaintiff narratives, are publicly available in NC, all identifying information has been removed from quotations.

### Types of Nonphysical Violence

#### Harassment and Stalking

“Harassment and Stalking” was characterized by constant unwanted calls and texts, loitering near or entering the plaintiff's residence uninvited, and frequenting the plaintiff's place of work or other locations where the plaintiff might be. In the following example, the plaintiff describes constant unwanted communication by the defendant. “[Defendant] has been harassing me with emails, phone calls, texts, for about 3 weeks. We are not together anymore, and I started dating.” In another example, a plaintiff describes harassment and stalking at her workplace, and the defendant's specific intentions of getting the plaintiff fired from her job.

He continuously harasses me by sending me numerous emails. I had to block him from social media and change my phone number because he would not stop harassing me. He went to my job numerous times when he had been told by management not to show up anymore. He went to my job on [dates redacted]. [Defendant] also sent harassing emails to my job stating lies trying to get me fired.

Causing interruptions at a plaintiff's place of work, through harassment and stalking, was also reflected in the following narrative in which the defendant not only trespasses at the plaintiff's place of work, but also hides a tracking device on the plaintiff's car.

I found a tracking device on my car and I’ve taken pictures of it. He has been showing up places that I go such as Walmart and the grocery store. On [dates redacted] he came to my workplace, even when I have repeatedly asked him not to. He will sit in his car and in my work parking lot and sometimes come up to my car. He leaves random notes all over my car every day. I will be driving and see him behind me and then suddenly he will be knocking on my window.

As demonstrated throughout the narratives, harassment and stalking manifested in diverse ways and had important implications on plaintiffs’ ability to go about their daily lives.

#### Degradation

Degradation includes situations in which the defendant curses at, disrespects, mocks, or insults the plaintiff in public or private settings. Private settings included the home or other settings in which only the plaintiff and the defendant were present. As demonstrated in the following example, the plaintiff describes the defendant's degrading behaviors occurring in the privacy of their bedroom, “About 30 minutes later he came into the bedroom and started to insult me calling [me] a ‘bitch’ and a ‘damned whore.’” In another example of private degradation, verbal abuse occurred during a car ride after the defendant misses a turn, “When I told him that he missed the turn, he started to yell and insult me. He would call me names like ‘piece of shit’ [and] ‘go fuck yourself.’ He continued to yell saying that he was not going to do whatever the ‘fuck’ I wanted.”

While the previous examples reflect how private degradation was used by defendants, degradation also occurred in public spaces. Among the narratives analyzed, public degradation was most often performed through social media. In the following quote the plaintiff describes the defendant's use of Facebook to inflict abuse, “[Defendant] slandered [Plaintiff] on Facebook stating ‘[Plaintiff] is a crack head, save her daughter from her’ and more … [Defendant] tagged me in a Facebook post that stated ‘[Plaintiff] is a walking corpse.’”

In another example of public degradation, the defendant uploads videos of his physical violence against the plaintiff to YouTube, “Since then he has been taunting and making secret recordings of me and has posted them on YouTube (spitting, shoving, pinning me down).” This example also highlights how a defendant used public degradation, a form of nonphysical violence, in conjunction with previous instances of physical violence.

#### Threats

Throughout the narratives, plaintiffs also described defendant's use of threats which fell into two categories: threats of suicide and threats to injure or kill the plaintiff and/or her loved ones. Regarding threats of suicide, defendants often made these claims when the plaintiff tried to end the relationship. For example, in the following narrative, the plaintiff describes attempting to end their marriage and the defendant retaliating by detaining the plaintiff in their car, while he threatened to kill himself, “I attempted to end the marriage; [dates redacted] Defendant had me drive while he held a gun saying he would kill himself.”

In another example, a defendant takes his threats a step further and actually attempts suicide, “Defendant attempted to commit suicide … because I would not reconcile the relationship.” In a final, more extreme example, the plaintiff describes finding the defendant hanging in their closest after an apparent attempted suicide. In this example, the plaintiff was not planning to leave the relationship but was instead trying to flee a violent situation in which her child was present.

So in front of our child he approached me with the pan, held up his arm, and said “I should hit you with this pan”. So I began to leave, he kept threatened to kill himself, 5 min later I found him [hanging in closet after apparent suicide attempt].

As demonstrated by these three excerpts, the threat or attempt of suicide was often characterized by the defendant's retaliation against a plaintiff's assertion of autonomy.

Beyond threats of self-harm, defendants also threatened to injure or kill the plaintiff. In some situations, these threats were described as punishments for seeking help.

He has said if I report him to the police he will come back and hurt me. And he told me “who are you calling, are you calling the police, because I am going to hit you and then you call them.”

In other situations, threats to injure and kill were directed not only at the plaintiff but also at her family, friends, and other bystanders.

Stating that he would beat my ass and cause harm to my family and he has also verbally stated that he would come to my residence, jobs, church, wherever I am and cause me bodily harm and anyone else who decides to get in it.

When defendants threatened to harm plaintiffs with weapons, they commonly threatened the use of a firearm. While some defendants would simply state their plans to shoot the plaintiff, “My husband [Defendant], communicated to me and posted on Facebook that he (Defendant) was going to fucking kill me and was going to shoot me in the head with a gun.” Other defendants not only threatened firearm use, but also insinuated that they might be in possession of a said firearm, increasing the level of intimidation, “the defendant came to my house on Friday, [date redacted] and said he was going to kill me. He also patted his pocked like he had a gun.”

#### Effects of Nonphysical Violence

Most commonly plaintiffs indicated that nonphysical violence had a significant impact on their mental health, and many cited fear as being the most immediate effect. The unpredictability of defendants’ behaviors, particularly around stalking and harassment, produced substantial fear for plaintiffs; in one example, a plaintiff describes that her fear is a direct result of the harassing and stalking behaviors of the defendant, “This makes me afraid, I never know when he's going to show up.” In another example of the connection between stalking and fear, a plaintiff describes that her fear is a result of the defendant's behaviors and his ability to maintain knowledge of her whereabouts despite her attempts to evade him, “He will not leave us alone and I need the courts to help. I have fled from him many times and he continues to find our address and I am afraid.”

We also found that these reactions had important consequences for the physical safety of plaintiffs. In some cases, the fear caused by the experience of nonphysical violence was strong enough to motivate plaintiffs to participate in behaviors that jeopardized their immediate physical safety. For example, a plaintiff described jumping out of a moving vehicle to flee her abuser out of fear.

He started to tell me to get out of the car while it was moving. I told him that I could not get out because he was still driving … then he [barely] came to a stop, but when I started to get out, he sped up and drove away. I lost my balance and almost fell. I started to walk but it hurt really bad … I fear this man.

In another example, a plaintiff described holding a gun to her own head after being instructed to do so by her abuser. She feared that if she did not comply with his demands, she would experience severe physical harm, possibly death

On [date redacted] he came to our house and made me put this gun to my head and I did it because he was very irate and demanding. I was afraid if I didn't he would beat me up or even kill me.

Overall, plaintiff's fear seemed related to feelings of uncertainty regarding the potential actions of defendants as well as the possibility of future harm. In some cases, this emotional response was strong enough to motivate plaintiffs to engage in physically dangerous behaviors in an attempt to placate the defendant.

## Discussion

By examining DVPO narratives, we found that nonphysical IPV is more prevalent than physical violence among DVPO plaintiffs. We identified three types of nonphysical violence that were commonly experienced by DVPO plaintiffs: (1) harassment and stalking; (2) degradation; and (3) threats. These findings are consistent with results from the National Intimate Partner and Sexual Violence Survey (2015) regarding types of stalking and psychological abuse that survivors experience (Smith et al., 2018). Additionally, several of the types of nonphysical violence frequently described by plaintiffs (e.g., threatening suicide, stalking, controlling behaviors, etc.) are components of lethality assessment protocols, as they have been linked to increased risk of femicide (Smith et al., 2017). Beyond identifying common types of nonphysical violence, we also found that nonphysical violence caused plaintiffs to experience fear, which sometimes created physically dangerous situations.

### Negative Consequences of Nonphysical Violence

As described in plaintiff narratives, defendants’ violent actions produced fear, which in some circumstances, precipitated behaviors by the plaintiff, such as holding a gun to her own head or jumping out of a moving vehicle that seemingly created a physically dangerous situation. Though these fear-driven behaviors seem counterintuitive, the empirical literature indicates that these apparently “self-harming” behaviors are actually quite rational and common among survivors of violence faced with the immediate threat of their abusers’ unpredictable behavior. For example, in their qualitative study, Short et al. (2000) found that fear was a driving factor for survivors of IPV's willingness to stay in violent relationships and that decisions to stay were “highly rational [choices] that carefully and accurately took into account the pros and cons of the situation” (Short et al., 2000). Much like what was expressed in plaintiff narratives analyzed for our study, Short et al. illustrate that while staying in a violent relationship often resulted in immediate bodily harm, study participants were motivated to risk this immediate danger out of fear of their abuser's more severe, future, violent behaviors if they attempted to leave (Short et al., 2000). They also found that survivor's fear was greatly influenced by acts of nonphysical violence such as threats (Short et al., 2000). Through creating fear via nonphysically violent behaviors, abusers maintained control and power over their victims; allowing for the precipitation of physically dangerous situations with the potential to result in death or other life-altering harms.

Sustained experiences of fear also have significant long-term implications for the mental health of IPV survivors. For example, in their survey of 220 IPV survivors, Salcioglu et al. (2017) determined that anticipatory fear, “due to an ongoing threat of safety, regarding their relationship with their abuser,” was the strongest predictor of posttraumatic stress disorder (PTSD) and depression symptomatology among participants (Salcioglu et al., 2017). While our study dataset does not include information regarding the long-term health outcomes of plaintiffs, as previously mentioned, plaintiffs do describe sustained fear as a frequent outcome of their abusive partner's behavior. Therefore, DVPO plaintiffs may be more susceptible to future poor mental health outcomes due to their sustained exposure to fear.

Beyond the consequences specific to fear, there is a well-established link between exposure to nonphysical violence and PTSD symptomatology (Golding, 1999; Logan et al., 2006)). Nonphysical acts of violence such as stalking and harassment are strongly associated with lifetime PTSD outcomes (Logan et al., 2006). Additionally, other manifestations of nonphysical violence, such as threats, also have significant mental health consequences for survivors; some survivors even cite their experiences of nonphysical violence as having had a more profound impact on their long-term quality of life than experiences of physical violence (O’Leary, 1999; Queen et al., 2009). Specifically relating to firearms and their threat of use by defendants, previous studies have shown that the threat of firearm use is a significant predictor of PTSD symptomatology, even when controlling for age and other psychological factors (Sullivan & Weiss, 2017). Additionally, like other forms of nonphysical IPV, survivors who experience threat of firearm use also express experiencing an abundance of fear (Adhia et al., 2021). Understanding this link between nonphysical IPV, fear, and long-term mental health outcomes reemphasizes the necessity of a thorough consideration of nonphysical IPV in DVPO cases.

### Recommendations to Improve NC DVPO Procedures

Based on our findings, we developed recommendations that can be implemented at the district court level to enhance consideration of nonphysical violence in the DVPO process. The aim of these recommendations is to facilitate a process in which judges can better identify and address the significance of plaintiff exposure to nonphysical IPV in their decision-making. As supported by both the established literature and the findings from the present study, we recognize that experiences of nonphysical violence have profound, negative, long- and short-term outcomes for IPV survivors. We also identified the ways in which these experiences might be associated with risky plaintiff behaviors and poor mental health outcomes.

It is imperative that judges carefully consider instances of nonphysical IPV in DVPO cases and the potential risk that it poses to the health and safety of the plaintiff. Therefore, we recommend the development of reference tools, such as a brief bench card, for judges to use when reviewing plaintiffs’ complaints before a DVPO hearing. This informational tool would highlight the common forms of nonphysical IPV to be taken into consideration when making DVPO determinations. We found that DVPO complaint narratives are a rich source of information that could contribute to DVPO decision-making, yet this critical information may be hard to locate within the relatively long DVPO complaint form. This could cause judges to spend precious moments looking for the narratives instead of being able to read and review the case. By moving the plaintiff’s complaints to a prominent location on the first page of the form, judges could more easily locate these important narratives, allowing them additional time to consider the entirety of the case when making decisions. We, therefore, recommend moving the plaintiff narratives to first pages of the complaint form.

In addition, changes in filing methods that would provide more informational support to plaintiffs could facilitate the consistent provision of pertinent information to judges, allowing them to make more well-informed decisions in DVPO cases. For example, currently, 14 counties in NC have implemented electronic filing (e-filing) for DVPOs. Plaintiffs who file electronically are guided through the process with the assistance of a DV advocate or court-appointed personnel. Furthermore, e-filed DVPO complaints are typed, rather than handwritten. Depending on education level, personal stress, distractions, fear, and a myriad of other unpredictable factors that may occur when plaintiffs are filing a DVPO complaint, handwritten narratives may be difficult to read and interpret. Our team found that e-filed cases tended to have a much more logical and chronological flow, highlighting the relevant experiences of violence that prompted the pursuit of the DVPO in detail. Overall, DVPO e-filing may be an effective means for improving the content, quality, and consistency of DVPO complaints, which would allow plaintiffs to share their stories with judges more readily, regardless of socio-demographic factors that may have previously impacted the readability and comprehensiveness of their complaint. The expansion of e-filing could also benefit judges as cases would require less time to read. Judges would have more time to consider the merits of the case instead of spending time attempting to decipher illegible handwriting, spelling mistakes, or grammatical errors.

Our research team is the first to use DVPO complaint form narratives as a source of information about plaintiffs’ experiences with nonphysical violence. We found these narratives to be an unexpectedly rich source of information, and we encourage other researchers to explore the utility of this data source. Additionally, future analyses of narratives should consider themes around physical and nonphysical violence as well as other judge-indicated areas of importance and how these factors correlate to granting and denial rates in DVPO cases.

### Sample Diversity and Study Limitations

While our study sample reflected racial and ethnic diversity, we understand that diversity extends beyond racial and ethnic identity and includes other demographic factors such as gender, sexual orientation, socioeconomic status, religion, rurality, etc. However, with our data source, we were not able to obtain all of this demographic information for litigants, limiting our ability to assess the full extent of our sample's diversity. As such, future research should specifically consider the ways that other demographic characteristics might relate to different manifestations of and experiences with nonphysical IPV. While qualitative analyses are not intended to be generalizable across all populations, such findings could bolster the conclusions of this study, indicating that nonphysical violence manifests similarly across demographic groups, or identify the nuanced ways that nonphysical violence varies depending on demographic contextual factors. DVPO processes are similar across all 50 states and the District of Columbia. Therefore, research regarding the experiences of diverse populations could help to identify ways in which these processes can be improved nationally; our study is the first of what we hope to be many projects that contribute to this important body of research.

Finally, most DVPO filing forms are structured such that the plaintiff is prompted to highlight a single, specific incident of violence that precipitated their filing for a DVPO (Agnew-Brune et al., 2017). However, due to the often chronic nature of IPV (Rand & Saltzman, 2003), it is possible that the plaintiffs excluded information about other experiences of IPV that were not directly related to the precipitating incident for which they were requesting the DVPO. This could potentially limit our understanding of the entirety of a plaintiff's experience with nonphysical IPV.

## Conclusion

Our study findings and subsequent recommendations indicate potential changes to facilitate access to information for judges when analyzing DVPO case files. It is possible that judges are not considering the severity of nonphysical manifestations of IPV simply due to time constraints and limited information, thus impacting their decision-making during DVPO hearings. Ultimately, these administrative changes might allow judges to identify critical acts of nonphysical IPV with important implications for the long-term health and safety of plaintiffs, thus improving their access to meaningful justice.
